# Dietary Pattern and Risk of Thyroid Cancer: A Systematic Review

**DOI:** 10.1111/jhn.70337

**Published:** 2026-08-25

**Authors:** Yasmin G. Nagashima, Aline A. Soares, Grasiela Piuvezam, Camila X. Alves, Kleyton S. Medeiros, José Brandão‐Neto, Márcia M. G. D. Lopes

**Affiliations:** ^1^ Postgraduate Programme in Health Sciences Federal University of Rio Grande do Norte Natal Brazil; ^2^ Institute of Education, Research and Innovation League Against Cancer Natal Brazil; ^3^ Systematic Review and Meta‐Analysis Laboratory (Lab‐Sys/CNPq) Federal University of Rio Grande do Norte Natal Brazil; ^4^ Monsignor Walfredo Gurgel Hospital Natal Brazil; ^5^ Department of Nutrition Federal University of Rio Grande do Norte Natal Brazil

**Keywords:** diet, dietary pattern, risk factors, systematic review, thyroid neoplasms

## Abstract

**Background:**

The incidence of thyroid cancer (TC) has significantly increased over the past decades due to a few recognised risk factors. Although the relationship between dietary factors and TC is still not fully understood, emerging evidence suggests that certain dietary habits may influence the risk of developing this type of cancer. This systematic review (PROSPERO ID: CRD42023463802) investigated the association between dietary patterns and TC risk using the available evidence.

**Methods:**

Five databases were searched (PubMed/Medline, Embase, Scopus, Web of Science and LILACS) for observational studies in adults comparing unhealthy and healthy dietary patterns among individuals diagnosed with TC—between September 2023 and January 2025, without restrictions on language or publication year. Three independent reviewers conducted study selection and data extraction.

**Results:**

Forty‐five studies were included (two cross‐sectional, thirteen cohort and thirty case‐control). *A posteriori* patterns rich in vegetables, fruits, fish, lean meat, dairy products and unsaturated fats were significantly associated with reduced TC risk, whereas starchy foods, seafood, seaweed, processed meats, instant foods, fast food and Western patterns increased risk. *A priori* patterns with higher inflammatory potential were also positively associated with TC risk. Most studies demonstrated good methodological quality, according to NOS assessment.

**Conclusion:**

This systematic review suggests that dietary patterns may be associated with TC risk. Although healthier dietary patterns tended to be associated with a lower risk and pro‐inflammatory or Western‐style dietary patterns with a higher risk, the current evidence remains observational and heterogeneous. Further prospective studies are needed to clarify the biological mechanisms underlying these associations.

## Summary

- Dietary patterns rich in vegetables, fruits, fish, lean meats, dairy products and unsaturated fats tended to be associated with a lower risk of thyroid cancer, whereas Western‐style, high‐glycaemic, processed‐meat and starchy‐food patterns tended to be associated with a higher risk.
- Evidence from the included studies suggests that dietary patterns with higher inflammatory potential (e.g., higher DII/E‐DII scores) may be associated with a higher risk of thyroid cancer.
- Current evidence remains predominantly observational and heterogeneous; therefore, findings should be interpreted with caution when considering thyroid cancer‐specific dietary recommendations.

## 1 Introduction

Thyroid cancer (TC) develops from the uncontrolled growth of follicular cells and represents the ninth most common endocrine and neuroendocrine malignancy worldwide [1, 2]. Future projections estimate a 12.2% global increase in TC cases [3]. In Brazil, a developing country, approximately 16,660 new cases are expected annually during 2023–2025 [4]. Among the histological subtypes, papillary carcinoma is the most frequent, followed by follicular carcinoma [5].

The rising incidence of TC may partly reflect advances in detection, as diagnostic methods have become more precise and accessible [2]. However, environmental exposures, lifestyle factors and comorbidities also play an important role [6, 7, 8]. Although the exact mechanisms responsible for TC development remain unclear, individuals with specific risk factors are more susceptible. The most established predictors are largely non‐modifiable, such as age, sex, ethnicity and family history [5].

Interactions between diet, lifestyle and environmental pollution may also contribute to TC risk [7]. Despite some contradictory and inconclusive findings regarding specific dietary components, the cumulative evidence, on the other hand, supports the hypothesis that both exposure to environmental pollutants and modifiable factors—particularly dietary habits—may influence TC development [9]. Previous research has reported associations between certain foods and TC, showing that some dietary factors may grant a protective effect, while others may increase risk [10, 11, 12]. However, it is crucial to consider the complex interactions and combined effects of different foods, nutrients, and dietary components, since their combined impact is greater than the effect of individual items [13].

From this perspective, dietary pattern analysis provides a broader understanding of the relationship between diet and disease, overcoming the limitations of assessing isolated foods or nutrients in relation to cancer risk [8].

Evaluating habitual dietary patterns can produce effective strategies and generate evidence more easily implementable in dietary guidelines [14].

Dietary patterns, defined as the set or grouping of foods typically consumed by a given population and assessed through dietary intake [15], may provide more robust effect estimates. They can be identified using *a posteriori* method, in which patterns emerge through statistical analysis of nutritional data. These methods, however, are likely to result in measurement errors that may bias associations between diet and health [16]. Alternatively, the *a priori* method uses established dietary patterns derived from dietary guidelines and specific indices [16]. Whereas this facilitates comparison across studies, significant variations may occur based on the selected components and adherence criteria, requiring careful interpretation when synthesising findings from various studies. In both approaches, dietary patterns are usually assessed using food frequency questionnaires (FFQs), designed to capture habitual consumption [14].

There is growing evidence that dietary patterns may influence TC carcinogenesis as well as its aggressiveness and severity [7, 8, 17]. For example, diets with higher inflammatory potential—indicated by elevated scores on the Energy‐adjusted Dietary Inflammatory Index (E‐DII), which often reflects high intake of processed foods and saturated fats—have been associated with increased TC risk [11]. In contrast, combined intake of iodine‐rich foods (such as seaweed, mussels and shrimp skin) has been linked to reduced risk compared with no intake [12]. However, findings are inconsistent, likely due to variability in dietary patterns, lifestyle and environmental exposures across different ethnic groups [18].

Recent studies have extensively investigated the relationship between three dietary groups—iodine‐rich foods, dairy products and starchy foods —with mixed results. Some reported [19, 20, 21] a positive association between excessive consumption of dairy and refined carbohydrates and TC risk, whereas others found no significant correlation [22, 23].

Although foods and beverages consumed daily contain a wide variety of constituents, their specific roles remain poorly understood [10], particularly regarding nutrition's impact on thyroid homeostasis and overall metabolic status [8]. Moreover, the biological mechanisms linking dietary patterns and TC risk are likely explained by synergistic or additive effects of multiple dietary components [14]. Recognising diet quality as a potentially modifiable factor may contribute to future guidance aimed at TC prevention in both men and women [12].

Considering that dietary patterns act synergistically to influence the risk of various chronic diseases [24] and that understanding diet–health connections is key to addressing current knowledge gaps, this systematic review aimed to investigate the scientific evidence on the relationship between dietary patterns and the risk of TC.

## 2 Methods

This systematic review of the literature was conducted in accordance with the Cochrane Handbook for Systematic Reviews of Interventions [25], following the reporting standards outlined by Preferred Reporting Items for Systematic reviews and Meta‐Analyses (PRISMA) [26, 27]. A protocol was developed prior to the review [28], and the systematic review was prospectively registered in PROSPERO (ID: CRD42023463802).

### 2.1 Search Strategy

The literature search was conducted between September 2023 and January 2025 in the following electronic databases: PubMed/MEDLINE, Embase, Scopus, Web of Science and LILACS. In addition, the reference lists of all included studies were manually screened to identify any potentially eligible articles not captured by the electronic search.

Following completion of the initial search, we observed that the number of eligible studies was limited, particularly for specific subtopics (e.g., *a priori* inflammatory indices). Therefore, a complementary search was conducted by incorporating additional search terms while maintaining the same eligibility criteria, databases and screening procedures as the initial search. This complementary search was developed after completion of the initial search to improve the sensitivity of the strategy and ensure that all relevant studies on dietary patterns and TC were identified. Studies identified through both the initial and complementary searches were screened using the same eligibility criteria and incorporated into the review. To ensure transparency and facilitate reproducibility, both complete search strategies are provided in the Supplementary Material (see Supplementary Table S1).

### 2.2 Inclusion Criteria

Inclusion criteria were observational studies (cohort, cross‐sectional, and case‐control) involving adults (> 18 years) diagnosed with TC, assessing the association between *a priori* or *a posteriori* dietary patterns and TC risk. No restrictions were applied regarding publication date or language. Case reports, meeting abstracts, review articles, book chapters, theses, and commentaries were excluded.

### 2.3 Study Selection

**Figure 1:**
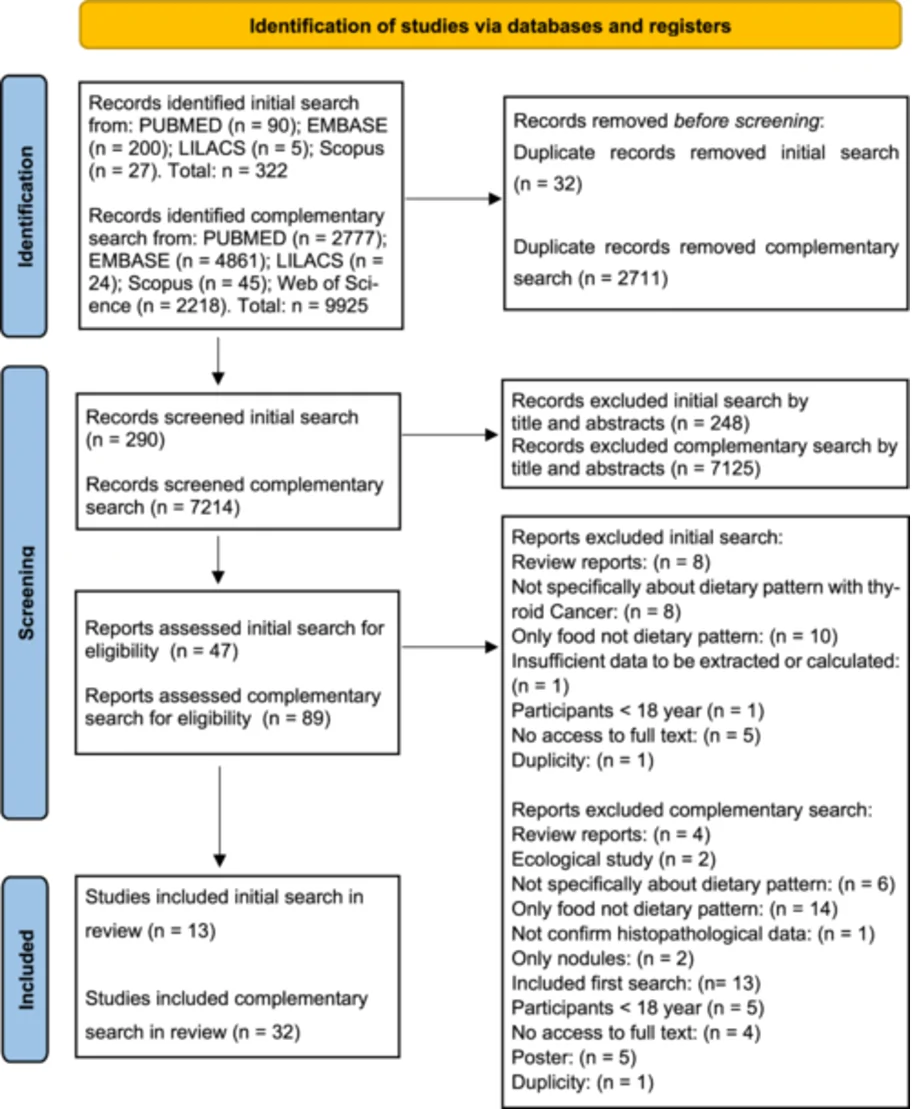
PRISMA flow chart of the literature search and study selection for dietary patterns and thyroid cancer; PRISMA, Preferred Reporting Items for Systematic Reviews and Meta‐Analyses.

Studies were independently screened by two reviewers, with disagreements resolved by a third reviewer. Screening of titles and abstracts was conducted using Rayyan (https://www.rayyan.ai). Duplicates and reviews were removed. Full‐text screening was performed to confirm eligibility. Study selection is summarised in the PRISMA flow diagram (Figure 1).

### 2.4 Data Extraction

Following the same procedure as study selection, data extraction was performed independently using a predesigned extraction form (see Supplementary Table S2). Extracted information included the first author, year of publication, country, study design, main objectives, study population, control sample, study duration and dietary pattern. Data was then compiled into tables to facilitate comparative analysis.

### 2.5 Methodological Quality

Risk of bias was independently assessed by two reviewers. The Newcastle‐Ottawa Quality Assessment Scale (NOS) was used to evaluate the methodological quality of the studies, and its adapted version was employed for cross‐sectional studies [29, 30].

## 3 Results

### 3.1 Search and Study Selection

The initial search identified 322 records. Following refinement of the search strategy through the incorporation of additional search terms, the complementary search retrieved an additional 9925 records. *Rayyan* detected 32 duplicates from the initial search and 2711 duplicates from the complementary search. From the initial search, 239 records were excluded after title and abstract screening (primarily due to irrelevance to dietary patterns or TC risk), and 13 full‐text publications were retained [19, 22, 23, 31, 32, 33, 34, 35, 36, 37, 38, 39, 40]. From the complementary search, 7125 records were excluded (studies not meeting the inclusion criteria for dietary patterns and TC risk), with 32 publications being included after applying eligibility criteria [11, 12, 20, 21, 41, 42, 43, 44, 45, 46, 47, 48, 49, 50, 51, 52, 53, 54, 55, 56, 57, 58, 59, 60, 61, 62, 63, 64, 65, 66, 67, 68]. Four additional studies were excluded because full texts could not be obtained despite contacting the authors, resulting in 45 studies being included in this review (Figure 1).

Study designs included observational designs, case‐control, cross‐sectional and both retrospective and prospective cohorts, evaluating the relationship between dietary patterns (*a priori* and *a posteriori*) and TC risk.

### 3.2 Study Characteristics

Eighteen studies were conducted in Europe, 17 in Asia, 7 in the Americas and 3 in Oceania (see Supplementary Figure S1). The included studies consisted of two cross‐sectional studies [55, 56], 13 cohort studies [19, 20, 23, 41, 44, 45, 47, 48, 51, 53, 59, 62, 63], 2 [44, 63] of which analysed the same population with distinct outcomes, and 30 case‐control studies [11, 12, 21, 31, 32, 33, 34, 35, 36, 37, 38, 39, 40, 41, 42, 43, 46, 49, 50, 52, 57, 58, 59, 64, 65, 66, 67, 68]. Among these, 18 were population‐based and 12 hospital‐based. Publication years ranged from 1989 to 2024.

A summary of study characteristics and main findings is presented in Table 1. Of the 45 included studies, 22 declared no conflicts of interest. *A posteriori* dietary patterns were investigated in 40 studies [19, 20, 21, 22, 23, 31, 32, 33, 34, 35, 36, 37, 38, 39, 40, 41, 42, 46, 47, 48, 49, 50, 51, 52, 53, 54, 55, 56, 57, 58, 59, 60, 61, 62, 63, 64, 65, 66, 67, 68], whereas five studies [11, 12, 43, 44, 45] adopted *a priori* approach associating it with the risk of TC. Most studies involved predominantly female participants, with average ages ranging from 36 to 63 years. This predominant focus on female participants is noteworthy, with nine studies exclusively including women, highlighting a potential gender‐specific emphasis in the literature that may reflect the higher incidence of TC in women [21, 36, 37, 41, 45, 55, 57, 66, 67].

**Table 1:** Summary of the characteristics and main findings of studies reporting the association between dietary patterns defined a priori or a posteriori and the risk of thyroid cancer (*n* = 45).

First author, year, country	Study design	No. Cases/Controls	Population/Age (mean)	Dietary assessment tool (no. of items)	Analytical approach to dietary pattern	Dietary pattern	Main findings
Franceschi et al. (1989) [40], Italy	Case–control	245 cases 411 controls	62 men 183 women/45 years	FFQ^a^ (31)	*A posteriori*	High‐starch foods consumption	High consumption of bread, polenta, potatoes, cheese and butter was significantly associated with increased risk. Some individual foods, such as chicken, salami and sausages, were also associated with increased TC risk.
Kolonel et al. (1990) [46], Hawaii	Case–control	191 cases 441 controls	51 men 140 women (–)	FFQ (150)	*A posteriori*	Cruciferous vegetables consumption	High intake of goitrogenic vegetables was associated with increased TC risk.
Negri et al. (1991) [65], Italy	Case–control	120 cases 6.147 controls	39 men 81 women/(–)	FFQ (37)	*A posteriori*	Fruit consumption	No association between fruit consumption and TC risk was identified.
Glattre et al. (1992) [58], Norway	Case–control	92 cases 460 controls	Not stratified by sex/(–)	FFQ (60) + 24 h recall^b^	*A posteriori*	Seafood consumption	Shellfish intake was associated with increased TC risk.
Wingren et al. (1993) [52], Switzerland	Case–control	248 cases 496 controls	43 men 205 women/(–)	FFQ (–)	*A posteriori*	Cruciferous vegetables/seafood consumption	Cruciferous vegetable consumption was associated with decreased TC risk. Seafood (fish, shellfish) was identified as protective.
Takezaki et al. (1996) [36], Japan	Case–control	94 cases 26.666 controls	94 women/(–)	FFQ (–)	*A posteriori*	Western/Japanese patterns	Coffee intake tended to be associated with reduced risk, whereas frequent consumption of green leafy vegetables tended to be associated with increased risk.
Galanti et al. (1997) [33], Sweden & Norway	Case–control	246 cases 440 controls	59 men 187 women/(–)	FFQ (18 Sweden e 19 Norway)	*A posteriori*	Cruciferous vegetables consumption	No protective effect of Brassica vegetables was observed.
Fioretti et al. (1999) [42], Italy	Case–control	399 cases 617 controls	108 men 291 women/44 years	FFQ (29)	*A posteriori*	Refined cereals consumption	No association with TC risk was identified.
Frentzel‐Beyme e Helmert. (2000) [49], Germany	Case–control	174 cases 348 controls	57 men 117 women/51 years	FFQ (47)	*A posteriori*	Green vegetables/Yellow vegetables consumption	Green vegetables have a protective effect. Some individual foods were also assessed: tomato, paprika, and coffee reduced TC risk.
Memon et al. (2002) [50], Kuwait	Case–control	313 cases 313 controls	75 men 238 women/36 years	FFQ (13)	*A posteriori*	Cruciferous vegetables consumption	High cruciferous vegetable intake was associated with increased TC risk. Some individual foods were also analysed: fresh fish showed a protective effect, while processed fish was positively associated with TC.
Horn‐Ross et al. (2002) [57], USA	Case–control	608 cases 558 controls	608 women/42 years	FFQ (–)	*A posteriori*	Fermented foods consumption	Consumption of traditional non‐fermented and non‐traditional soy‐based foods and sprouts was associated with reduced risk.
Markaki et al. (2003) [38], Greece	Case–control	113 cases 138 controls	31 men 82 women/41 years	FFQ (100)	*A posteriori*	Fish and cooked vegetables consumption	Consumption of fish and cooked vegetables was positively associated with disease risk.
Haselkorn et al. (2003) [66], USA	Case–control	608 cases 558 controls	608 women/(–)	FFQ (–)	*A posteriori*	Isoflavone intake	Low isoflavone intake combined with the presence of goitre/nodule increased TC incidence.
Randi et al. (2008) [21], Italy	Case–control	399 cases 617 controls	108 men 291 women/44 years	FFQ (29)	*A posteriori*	Glycemic index/Glycemic load	Both glycemic index and glycemic load were directly associated with TC risk.
Truong et al. (2010) [22], New Caledonia	Case–control	293 cases 354 controls	293 women/(–)	FFQ (110)	*A posteriori*	Cruciferous vegetables/Dairy products consumption	Suggested a positive association between cruciferous vegetable intake and TC risk. No association was found with dairy products.
Jung et al. (2012) [37], South Korea	Case–control	111 cases 111 controls	111 women/50 years	FFQ (–) + 24h recall	*A posteriori*	Raw vegetables consumption	Raw vegetable intake reduced TC risk. Some individual foods were also assessed: persimmons and tangerines showed a significant protective effect.
Przybylik‐Mazurek et al. (2012) [31], Poland	Case–control	284 cases 345 controls	30 men 254 women/55 years	FFQ (50)	*A posteriori*	Vegetables and fruits consumption	Vegetable and fruit intake showed a protective effect. Some individual foods were also assessed: saltwater fish (an iodine source) and lean meat may represent important protective factors.
Clero et al. (2012) [56], French Polynesia	Case–control	229 cases 371 controls	26 men 203 women/(–)	FFQ (66)	*A posteriori*	Fish and seafood consumption	High fish and seafood consumption was associated with decreased TC risk.
Yoo et al. (2016) [32], South Korea	Case–control	102 cases 115 controls	12 men 87 women/49 years	FFQ (15)	*A posteriori*	Instant food consumption	Intake of instant foods was associated with increased TC risk.
Sangsefidi et al. (2019) [35], Iran	Case–control	309 cases 268 controls	9 men 36 women/38 years	FFQ (160)	*A posteriori*	Western, Traditional, Transitional, Healthy patterns	Western dietary patterns were associated with higher TC risk compared with the other patterns.
Paquet et al. (2020) [11], New Caledonia	Case–control	332 cases 412 controls	39 men 293 women/(–)	FFQ (45)	*A priori*	Energy‐adjusted Dietary Inflammatory Index	Positive associations were observed between higher energy‐adjusted DII scores and TC risk.
Fiore et al. (2020) [34], Italy	Case–control	106 cases 217 controls	23 men 83 women/47 years	FFQ (–)	*A posteriori*	Starchy foods/Meat and derivatives consumption	High intake of starchy foods increased risk in women; meat and derivatives were also associated with increased risk. Some individual foods were assessed: wine, milk, and dairy products were associated with decreased TC risk.
Liang et al. (2020) [39], USA	Case–control	390 cases 436 controls	71 men 319 women/51 years	FFQ (148)	*A posteriori*	Fruit and vegetable consumption	Fruit and vegetable intake had a strong protective effect, particularly in women aged ≥ 50 years.
Amiri et al. (2021) [60], Iran	Case–control	115 cases 230 controls	115 women/52 years	FFQ (–)	*A posteriori*	Fast food consumption	Fast food intake was identified as a predisposing factor for TC.
Fiore et al. (2021) [61] Italy	Case–control	106 cases 121 controls	23 men 83 women/52 years	FFQ (–)	*A posteriori*	Simple carbohydrate–rich foods consumption	Intake of simple carbohydrates was associated with increased TC risk.
Xia et al. (2021) [68], China	Case–control	402 cases 402 controls	119 men 283 women/41 years	FFQ (–)	*A posteriori*	Iodine‐rich foods consumption	Consumption of iodine‐rich foods was associated with a reduced risk of TC compared with non‐consumption.
Parad et al. (2021) [64], Iran	Case–control	361 cases 347 controls	59 men 302 women/44 years	FFQ (–)	*A posteriori*	Unsaturated versus saturated oils consumption	Intake of unsaturated oils showed a protective effect for TC compared with saturated oils.
Lécuyer et al. (2022) [43], France	Case–control	1321 cases 1502 controls	284 men 1037 women/51 years	FFQ (158) + FFQ (64)	*A priori*	Dietary Inflammatory Index, residual and density methods	Positive associations were observed between all three DII measures and TC risk.
Xia et al. (2023) [12], China	Case–control	414 cases 414 controls	111 men 303 women/(–)	FFQ (121)	*A priori*	Chinese Healthy Diet Index (CHDI)	Higher CHDI scores were associated with reduced TC risk.
Abdulaziz et al. (2023) [67], Saudi Arabia	Case–control	398 cases 100 controls	398 women/39 years	FFQ (–)	*A posteriori*	Vegetarian, Non‐vegetarian, Iodine‐supplemented vegetarian	Vegetarian diet without iodine supplementation was associated with iodine deficiency and increased TC risk.
Michikawa et al. (2012) [62], Japan	Cohort	134 cases52,679 total	134 women/52 years	FFQ (–)	*A posteriori*	Seaweed consumption	Intake of seaweed tended to be associated with increased TC risk. Seaweed consumers also consumed fish, green vegetables, potatoes, pickled vegetables and soy products.
Aschebrook‐Kilfoy et al. (2012) [41], China	Cohort	164 cases 73,317 total	164 women/50 years	FFQ (77)	*A posteriori*	Nitrite‐ and nitrate‐rich foods consumption	Intake of processed meats (high in nitrite) was associated with increased TC risk.
Xiao et al. (2014) [53], USA	Cohort	586 cases 49,1840 total	Not stratified by sex/ (–)	FFQ (–)	*A posteriori*	Flavonoid consumption	Total flavonoid intake (tea, citrus fruits and juices, legumes, other fruits and vegetables) was associated with reduced TC risk.
Wie et al. (2014) [59], Korea	Cohort	136 cases 7637 total	64 men 72 women/49 years	Dietary record 3 days	*A posteriori*	Seaweed consumption	No statistically significant association between seaweed consumption and TC risk.
Zamora‐Ros et al. (2018) [51], Europe	Cohort	748 cases 476,108 total	12 men 87 women/(–)	FFQ (–)	*A posteriori*	Fruit and vegetable consumption	No association was observed between total fruit and vegetable intake and TC risk.
Tanitame et al. (2022) [23], Japan	Cohort	190 cases 99,634 total	29 men 161 women/(–)	FFQ (45)	*A posteriori*	Dairy product consumption	Intake of milk and dairy products was associated with reduced TC risk in women with BMI ≥ 25.0 kg/m^2^.
Llaha et al. (2022) [45], Europe	Cohort	712 cases 450,064 total	712 women/51 years	FFQ (–)	*A priori*	Mediterranean diet	Adherence to the Mediterranean diet was not associated with TC risk.
Jin et al. (2022) [48], Europe	Cohort	649 cases 431 total	Not stratified by sex/(–)	FFQ (–)	*A posteriori*	Dried fruit consumption	No causal relationship was observed between dried fruit intake and TC risk.
Lécuyer et al. (2022) [44], Europe	Cohort	712 cases 450,063 total	71 men 641 women/51 years	FFQ (–)	*A priori*	Dietary Inflammatory Index, Residual method DII, Density method DII, Dietary Inflammatory Score	Weak positive associations were observed between all three indices and TC risk.
Nguyen et al. (2023) [19], South Korea	Cohort	138 cases 13,835 total	18 men 120 women/51 years	FFQ (–)	*A posteriori*	Dairy product consumption	Dairy intake showed a stronger protective effect in participants older than 50 years.
La Greca et al. (2023) [47], USA	Cohort	426 cases 304,122 total	Not stratified by sex/63 years	FFQ (124)	*A posteriori*	Vegetable group, Fruit group consumption	Higher vegetable intake was positively associated with TC. No clear trends were observed for fruit or fish intake regarding TC risk.
Zamora‐Ros et al. (2023) [63], Europe	Cohort	681 cases 450,064 total	75 men 606 women/50 years	FFQ (36)	*A posteriori*	High consumption of sugar‐sweetened beverages	Diets high in sugar‐sweetened beverages tended to be associated with increased TC risk.
Kwon et al. (2024) [20], Korea	Cohort	930 cases 168,127 total	75 men 855 women/53 years	FFQ (–)	*A posteriori*	Dairy products, Seaweed consumption	High dairy consumption was associated with lower TC risk, whereas higher seaweed intake was associated with lower TC prevalence.
Choi et al. (2021) [55], South Korea	Cross‐sectional	407 cases 5696 healthy	52 men 355 women/49 years	FFQ (106) + 24 h recall	*A posteriori*	Seaweed consumption	Excessive iodine intake was associated with higher TC risk.
Vasconcelos et al. (2022) [56], Brazil	Cross‐sectional	30 cases 32 benign disease	5 men 27 women/43 years	24 h recall	*A posteriori*	Sugar group consumption	Sugar intake was higher among individuals with TC compared to others.

^a^ FFQ, Food Frequency Questionnaire.

^b^ 24 h recall, 24‐hour dietary recalls.

The most common dietary assessment method was the FFQ, used in 40 studies. A Brazilian study [56] applied 24‐h recalls (24hR) on 2 weekdays and 1 weekend day, while a Korean study [59] used a 3‐day food record within the same week. Three studies [37, 55, 58] combined FFQ and 24 hR. The number of food items assessed varied between 18 and 160.

In 10 studies [31, 45, 48, 51, 52, 53, 58, 59, 62, 63], dietary assessments were self‐administered; one study [60] conducted both face‐to‐face and telephone interviews. Four studies [20, 47, 49, 67] did not clearly specify how data were collected, while the remaining used interviewer‐administered methods. Only one Greek study [38] was blinded, with interviewers unaware of disease status or study hypotheses. All dietary assessments were performed at a single time point.

Food composition databases varied by country, with some studies using validated national references and others relying on proprietary databases, while the Brazilian study [56] used the Brazilian Food Composition Table (TBCA). Most studies employed visual aids, such as colour photographs, to improve portion size estimation.

### 3.3 Risk of Bias Assessment

Methodological quality was evaluated using the NOS [29, 30], with scores ranging from 5 to 9 (see Supplementary Table S3). Most studies were classified as high quality. Only three studies [36, 59, 67] received moderate‐quality ratings due to inadequate control for key confounding factors; for instance, control selection based on convenience sampling, limited representativeness of the exposed cohort, outcome assessment through self‐report and exposure determination without a clear description. The two cross‐sectional studies [55, 56] were rated as satisfactory and good, respectively.

The most frequent methodological limitations were related to exposure assessment, specifically, non‐blinded interviews for case/control status due to the potential for interviewer bias in data collection and lack of reporting on response rates.

### 3.4 *A priori* Dietary Patterns

Of the five studies [11, 12, 43, 44, 45], three case‐control studies [11, 12, 43] and two cohort studies [44, 45] evaluated the association between *a priori* dietary patterns and the risk of TC. Three of these studies [11, 43, 44] specifically examined the inflammatory potential of the diet and its impact on TC using the following indices: Dietary Inflammatory Index (DII), E‐DII, Energy‐adjusted DII using the density method (E‐DIId), Adapted Dietary Inflammatory Index (ADII) and Inflammatory Score of the Diet (ISD). These validated indices use algorithms to score overall diet quality based on 45 dietary components, accounting for the entire diet rather than individual nutrients or foods. Higher scores indicate a pro‐inflammatory diet, characterised by high ingestion of processed foods, sugar, saturated fat, red meat and low antioxidant content [11, 43].

In the European Prospective Investigation into Cancer and Nutrition (EPIC) cohort [44], all three DII versions were positively associated with TC risk, whereas ISD showed no significant association. In the New Caledonia study [11], a similar positive relationship was observed for E‐DII. The study clarifies that ADII reflects an individual's dietary inflammatory potential rather than the specific inflammatory processes involved in TC aetiology. Within this context, Lécuyer et al. [43] reported positive associations for ADII in the overall population, which were more pronounced in women.

In the European study [45], adherence to the Mediterranean diet was not associated with TC risk. Diet components assessed included vegetables, fruits, cereals, olive oil, fish, meat, dairy and alcohol. In contrast, the Chinese study [12] examined the Chinese Healthy Diet Index (CHDI), based on the 2016 Chinese Dietary Guidelines and Food Pyramid and found that higher CHDI scores were associated with a reduced risk of TC in both sexes.

### 3.5 *A posteriori* Dietary Patterns

Forty studies [19, 20, 21, 22, 23, 31, 32, 33, 34, 35, 36, 37, 38, 39, 40, 41, 42, 46, 47, 48, 49, 50, 51, 52, 53, 54, 55, 56, 57, 58, 59, 60, 61, 62, 63, 64, 65, 66, 67, 68] investigated associations between *a posteriori* dietary patterns and TC risk. Among these, 14 studies [19, 20, 31, 34, 37, 49, 52, 53, 54, 55, 57, 64, 66, 68] identified dietary patterns that seemed protective, reducing TC risk. There was considerable variability in the food groups represented. Two Asian studies [19, 20] reported that dairy and dairy‐derived products significantly reduced TC risk. Interestingly, one study [19] noted that longer meal durations (> 10 min) were associated with lower TC risk.

Patterns including raw vegetables [37], cruciferous vegetables [49, 52], and combinations of fish, vegetables, milk and fruits [31, 34] were also associated with reduced TC risk. Studies [20, 54, 55, 68] indicated additional benefits from iodine‐rich foods, such as seaweed. However, it is crucial to note that excessive intake of seaweed, as highlighted in Michikawa et al. [62], has been linked to increased TC risk due to potential over‐exposure to iodine.

While ten patterns [22, 23, 39, 47, 48, 50, 51, 59, 62, 65] showed no overall relevant association with TC risk, some of these studies revealed important sub‐group specific findings. For instance, one U.S. study [39], when stratified by sex, found a positive association between a ‘starchy foods and desserts’ pattern in men and a negative association for the ‘fruits and vegetables’ pattern in women.

Among 17 unfavourable dietary patterns [21, 32, 33, 35, 36, 38, 40, 41, 42, 46, 56, 58, 60, 61, 62, 63, 67], high nitrite content [41] (processed meats), instant foods [32] and fast food [60] were linked to increased TC risk. Patterns high in starch [40], refined cereals [42, 61], and sweetened beverages [56, 63] were also associated with higher TC risk. The EPIC cohort [63] showed particularly strong associations with papillary TC.

A study among Saudi women [67] identified a vegetarian pattern as a potential risk factor for TC, specifically when consumed without adequate iodine supplementation, attributed to overall low iodine intake despite the inclusion of ‘dairy options’. Additional associations involved tumour size and mitochondrial DNA (mtDNA) mutations. Within the vegetarian pattern, tumours were significantly larger and mtDNA mutations more frequent compared with other dietary groups. No association was observed among vegetarians supplemented with iodine. The vegetarian pattern was defined as daily consumption of vegetables, fruits, dairy options, plant‐based protein sources, starch‐rich carbohydrates, beans, low‐fat foods and staple items low in sugar and salt, and the quantitative dietary assessment was not measured.

In contrast, the Western dietary pattern was linked to higher TC risk. Takezaki et al. [36] compared a Western breakfast (bread and coffee) with a Japanese breakfast (rice and miso soup), finding that regular consumption of the Western breakfast increased TC risk. Similarly, a study conducted in Iran [35] reported a significant association between the Western dietary pattern, including offal, canned fish, fried or roasted meat, cooked salad with mayonnaise, potatoes, high‐fat dairy, snacks, tea, coffee, sugar and nuts and increased TC risk.

Additionally, a study in Norway and Sweden [33] observed no protective effect of cruciferous vegetables, likely due to their low consumption by these populations. Limited intake complicated result interpretation, generating controversy regarding the association between cruciferous vegetable consumption and TC risk.

## 4 Discussion

In this systematic review, we present a narrative synthesis of the available epidemiological evidence on the association between *a priori* and *a posteriori* dietary patterns and the risk of TC, aiming to provide a comprehensive overview of the current evidence and identify patterns that may guide future research. Furthermore, our review focused on overall dietary patterns rather than individual dietary components. Therefore, studies evaluating isolated foods or alcohol consumption were excluded.

Of the 45 studies summarised in Table 1, five investigated *a priori* dietary patterns [11, 12, 43, 44, 45]. Although the evidence is limited and should be interpreted with caution, the available studies suggest that pro‐inflammatory dietary patterns may be associated with an increased risk of TC. One possible explanation is that the combined effects of dietary components with pro‐inflammatory properties may contribute to a higher inflammatory burden, which has been proposed as one potential mechanism linking diet and cancer development [11]. On the other hand, greater adherence to the CHDI [12] was associated with a lower risk of TC. Although this finding suggests that overall diet quality may be relevant in the context of TC risk, additional evidence is needed to better understand these associations.

Various *a posteriori* dietary patterns showed inconsistent associations across the included studies. For instance, cruciferous vegetable consumption was associated with a lower risk of TC in some studies, whereas others reported a higher risk of TC. One possible explanation for these divergent findings is the goitrogenic effect of cruciferous vegetables, which may reduce iodine absorption through thiocyanate formation, although differences in iodine intake, dietary assessment methods, and study populations may also have contributed to the observed heterogeneity [33]. Markaki et al. [38] reported that the ‘fruit’ pattern, characterised by high consumption of fruits (peaches, cherries, melon, apricots, pears, apples, tangerines, watermelon, grapes, oranges, strawberries, lemons and figs) and ‘raw vegetable’ pattern (cucumber, fresh onion, bell pepper, tomato and lettuce) was associated with a lower risk of papillary TC, whereas a dietary pattern characterised by ‘fish and cooked vegetables’ (six types of cooked vegetables and fish) was associated with a higher risk of follicular TC. A study conducted in Poland [31] reported similar findings, suggesting that dietary patterns rich in vegetables, fruits, saltwater fish (an important dietary source of iodine) and lean meats were associated with a lower risk of TC.

Concerning refined carbohydrates and sugary foods, six studies [34, 39, 40, 42, 61, 63] reported a positive association with TC risk. Several biological mechanisms have been proposed to explain these observed associations, particularly those related to dietary glycemic index and glycemic load. An Italian study [21] also reported a positive association between higher dietary glycemic index/load and TC risk. These findings are biologically compatible with hypotheses involving hyperglycaemia, insulin signalling and cancer‐related pathways; however, the observational evidence included in this review cannot establish whether these mechanisms explain the reported associations. Although these findings may have implications for dietary guidance, they should be interpreted with caution, as differences in dietary assessment methods, geographical regions and study populations may have contributed to the heterogeneity observed across studies.

Regarding vegetarian dietary patterns, one study conducted among Saudi women [67] reported an association between a vegetarian dietary pattern without iodine supplementation and a higher risk of TC. Given that this finding was derived from a single study conducted in a specific population with low iodine intake, it should be interpreted with caution and should not be generalised to other populations. Nevertheless, it highlights the potential importance of considering iodine intake when evaluating vegetarian dietary patterns in future epidemiological studies.

In summary, *a posteriori* dietary patterns characterised by higher consumption of starchy foods, seafood, Western dietary pattern, high glycemic index, iodine‐rich foods (seaweed), nitrite‐rich foods (processed meats), instant meals and fast food were associated with increased TC risk. In contrast, patterns rich in plant‐based foods, fruits, fish, lean meats, dairy products and unsaturated fats may be associated with a lower risk of TC. However, these findings were not consistent across populations and should be interpreted cautiously in light of the substantial methodological and clinical heterogeneity among the included studies. Evidence on *a priori* dietary patterns was more limited but relied on validated tools. Overall, diets with higher inflammatory potential were associated with a higher risk of TC, whereas higher‐quality dietary patterns tended to be associated with a lower risk of TC.

Taken together, the available evidence suggests that considering overall dietary patterns, rather than isolated food items, may provide a broader perspective on the relationship between diet and TC risk. This hypothesis is based on the premise that the overall combination of foods within a dietary pattern may be more relevant than the effects of individual dietary components [13]. Although healthy dietary patterns are broadly recommended for general health, the available evidence remains insufficient to support TC‐specific dietary recommendations [69].

Regarding the limitations of our study, we acknowledge that the heterogeneity in dietary pattern definitions, variations in sample size, differences in dietary assessment methods (FFQ, 24 h, and dietary records), iodine intake across populations, geographical regions and TC subtypes limited our ability to draw definitive conclusions regarding the association between dietary patterns and TC risk. The varying number of food items in FFQs (from 18 to 220 items) across studies may affect the precision and comprehensiveness of dietary pattern capture, leading to inconsistencies and limiting the strength of the overall conclusions.

In addition, the inability to conduct subtype‐specific analysis due to insufficient reported data in primary studies limits the clinical applicability and biological interpretability of our findings, thereby limiting the interpretation of dietary influences on specific TC etiologies.

On the other hand, one of the strengths of this review lies in updating the current evidence on the relationship between dietary patterns and TC. By considering dietary patterns as the exposure of interest, this review provides a more comprehensive representation of overall diet, allowing the assessment of combinations of foods and nutrients rather than isolated dietary components. Although the methodological quality of most included studies was rated as good or excellent according to the NOS, these ratings should be interpreted considering the inherent limitations of observational studies, particularly case‐control designs, including recall bias, residual confounding, selection bias, and exposure misclassification. Furthermore, the search strategy was developed in collaboration with a specialised librarian, contributing to the comprehensiveness of the literature search and the identification of the available evidence.

Further prospective studies are needed to better understand these associations. Future research should include repeated dietary assessments over time using validated instruments to capture changes in dietary patterns and better reflect cumulative exposure to minimise measurement bias, and, whenever feasible, ensure blinded outcome assessment. In addition, studies should aim to collect longitudinal data on lifestyle factors (smoking status, physical activity levels and alcohol consumption), environmental exposures (persistent organic pollutants and heavy metals), food intake and their potential influence on TC progression and histological subtypes. Additional high‐quality prospective studies are warranted to clarify these associations and reduce the uncertainty arising from the heterogeneity of the available evidence.

## 5 Conclusion

This review suggests that overall dietary patterns may be associated with TC risk. Dietary patterns characterised by higher inflammatory potential, refined carbohydrates and Western‐style foods were generally associated with a higher risk of TC, whereas patterns rich in plant‐based foods, lean proteins, dairy products and unsaturated fats were generally associated with a lower risk of TC. Although evidence on *a priori* dietary patterns remains limited, the available findings highlight the importance of considering overall dietary patterns, rather than isolated dietary components, when investigating dietary associations with TC risk. Given the observational nature of the available evidence and the heterogeneity across studies, these findings should be interpreted with caution. Additional well‐designed prospective studies incorporating repeated dietary assessments and improved control of potential confounding factors are needed to further clarify these associations.

## Author Contributions

Yasmin G. Nagashima conceived the study; Aline A. Soares, Camila X. Alves contributed to the protocol development, Yasmin G. Nagashima and Aline A. Soares screened search results and extracted data and conducted quality appraisals of included studies; Camila X. Alves resolved conflicts with study selection, data extraction and quality appraisal; Yasmin G. Nagashima and Aline A. Soares led data synthesis and draughting of the manuscript; Yasmin G. Nagashima, Aline A. Soares, Grasiela Piuvezam, Camila X. Alves, Kleyton S. Medeiros, José Brandão‐Neto and Márcia M. G. D. Lopes revised the manuscript; Márcia M. G. D. Lopes was responsible for supervision and approval of the final content; all authors read and approved the final manuscript.

## Funding

The authors have nothing to report.

## Conflicts of Interest

The authors declare no conflicts of interest.

## Supporting information


Supporting File 1

Supporting File 2

## Data Availability

Nutrition in Clinical Practice (NCP).
