# Supplementary material for: Dietary Pattern and Risk of Thyroid Cancer: A Systematic Review
Source: J Hum Nutr Diet. 2026 Aug 25;39(4):e70337. doi: 10.1111/jhn.70337 (PMC13507658; doi:10.1111/jhn.70337)
Supplement: Supplementary file 1 [file JHN-39-0-s002.pdf]

Figure S1. Distribution of thyroid cancer publications by continent and number of cases over time.

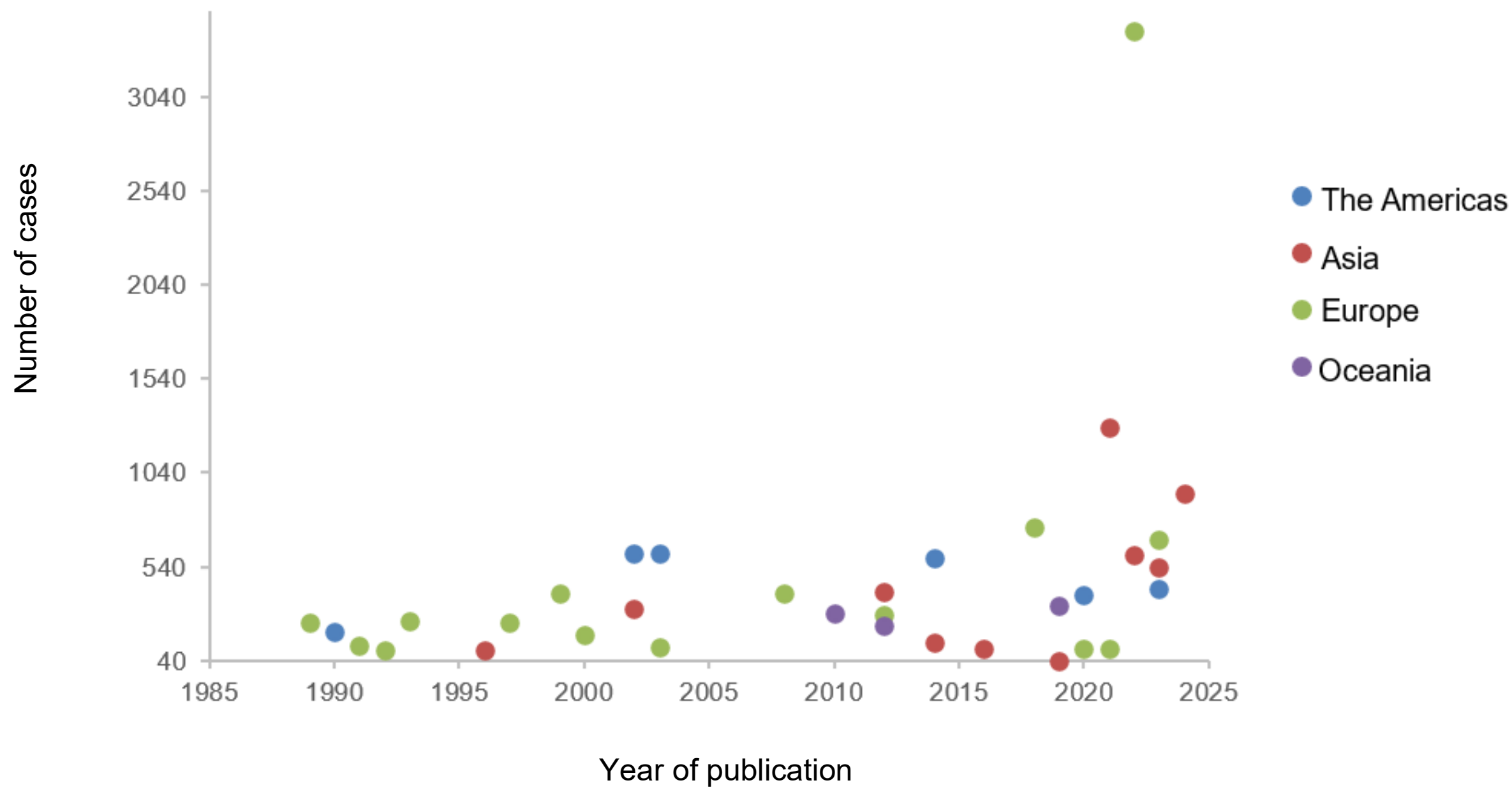

Source: Authors, 2025.
