# Supplementary material for: Dietary Pattern and Risk of Thyroid Cancer: A Systematic Review
Source: J Hum Nutr Diet. 2026 Aug 25;39(4):e70337. doi: 10.1111/jhn.70337 (PMC13507658; doi:10.1111/jhn.70337)
Supplement: Supplementary file 2 [file JHN-39-0-s001.docx]

**Supplementary Material**

**Supplementary Table S1 – Initial and complementary search strategies used in electronic databases on dietary patterns and thyroid cancer**

| ***Database*** | **Initial Search Strategy** | **Date** |
| --- | --- | --- |
| *Pubmed (Medline)* | *("Behavior, Eating" OR "Behavior, Feeding" OR "Behavior, Feeding-Related" OR "Behaviors, Eating" OR "Behaviors, Feeding" OR "Diet Habit" OR "Diet Habits" OR "Dietary Habit" OR "Dietary Habits" OR "Eating Behavior" OR "Eating Behaviors" OR "Eating Habit" OR "Eating Habits" OR "Feeding Behaviors" OR "Feeding Pattern" OR "Feeding Patterns" OR "Feeding Related Behavior" OR "Feeding-Related Behavior" OR "Feeding-Related Behaviors" OR "Food Habit" OR "Food Habits" OR "Habit, Diet" OR "Habit, Dietary" OR "Habit, Eating" OR "Habit, Food" OR "Habits, Diet" OR "Habits, Dietary" OR "Habits, Eating" OR "Habits, Food" OR "Pattern, Feeding" OR "Patterns, Feeding" OR "Diet, Meat-Sweet" OR "Diet, Occidental" OR "Dietary Pattern, Western" OR "Dietary Patterns, Western" OR "Diets, Meat-Sweet" OR "Diets, Occidental" OR "Diets, Western" OR "Meat Sweet Diet" OR "Meat-Sweet Diet" OR "Meat-Sweet Diets" OR "Occidental Diet" OR "Occidental Diets" OR "Pattern, Western Dietary" OR "Patterns, Western Dietary" OR "Western Diet" OR "Western Dietary Pattern" OR "Western Dietary Patterns" OR "Western Diets") AND ("Adenoma, Thyroid" OR "Adenomas, Thyroid" OR "Cancer of the Thyroid" OR "Cancer of Thyroid" OR "Cancer, Thyroid" OR "Cancers, Thyroid" OR "Carcinoma, Thyroid" OR "Carcinomas, Thyroid" OR "Neoplasm, Thyroid" OR "Neoplasms, Thyroid" OR "Radioiodine-Refractory Thyroid Cancer" OR "Thyroid Adenoma" OR "Thyroid Adenomas" OR "Thyroid Cancer" OR "Thyroid Cancers" OR "Thyroid Carcinoma" OR "Thyroid Carcinomas" OR "Thyroid Neoplasm")* | *10/09/2023* |
| *Embase (Elsevier)* | *('behavior, eating'/exp OR 'behavior, eating' OR 'behavior, feeding' OR 'behavior, feeding-related' OR 'behaviors, eating' OR 'behaviors, feeding' OR 'diet habit' OR 'diet habits' OR 'dietary habit'/exp OR 'dietary habit' OR 'dietary habits' OR 'eating behavior'/exp OR 'eating behavior' OR 'eating behaviors' OR 'eating habit'/exp OR 'eating habit' OR 'eating habits' OR 'feeding behaviors' OR 'feeding pattern'/exp OR 'feeding pattern' OR 'feeding patterns' OR 'feeding related behavior' OR 'feeding-related behavior' OR 'feeding-related behaviors' OR 'food habit'/exp OR 'food habit' OR 'food habits'/exp OR 'food habits' OR 'habit, diet' OR 'habit, dietary' OR 'habit, eating' OR 'habit, food' OR 'habits, diet' OR 'habits, dietary' OR 'habits, eating' OR 'habits, food' OR 'pattern, feeding' OR 'patterns, feeding' OR 'diet, meat-sweet' OR 'diet, occidental' OR 'dietary pattern, western' OR 'dietary patterns, western' OR 'diets, meat-sweet' OR 'diets, occidental' OR 'diets, western' OR 'meat sweet diet' OR 'meat-sweet diet' OR 'meat-sweet diets' OR 'occidental diet' OR 'occidental diets' OR 'pattern, western dietary' OR 'patterns, western dietary' OR 'western diet'/exp OR 'western diet' OR 'western dietary pattern'/exp OR 'western dietary pattern' OR 'western dietary patterns' OR 'western diets') AND ('adenoma, thyroid' OR 'adenomas, thyroid' OR 'cancer of the thyroid'/exp OR 'cancer of the thyroid' OR 'cancer of thyroid'/exp OR 'cancer of thyroid' OR 'cancer, thyroid' OR 'cancers, thyroid' OR 'carcinoma, thyroid' OR 'carcinomas, thyroid' OR 'neoplasm, thyroid' OR 'neoplasms, thyroid' OR 'radioiodine-refractory thyroid cancer' OR 'thyroid adenoma'/exp OR 'thyroid adenoma' OR 'thyroid adenomas' OR 'thyroid cancer'/exp OR 'thyroid cancer' OR 'thyroid cancers' OR 'thyroid carcinoma'/exp OR 'thyroid carcinoma' OR 'thyroid carcinomas' OR 'thyroid tumor'/exp OR 'thyroid tumor')* | *10/09/2023* |
| *Scopus (Elsevier)* | *‘Behavior Eating’ OR ‘Behavior Feeding’ OR ‘Behavior Feeding-Related’ OR ‘Behaviors Eating’ OR ‘Behaviors Feeding’ OR ‘Diet Habit’ OR ‘Diet Habits’ OR ‘Dietary Habit’ OR ‘Dietary Habits’ OR ‘Eating Behavior’ OR ‘Eating Behaviors’ OR ‘Eating Habit’ OR ‘Eating Habits’ OR ‘Feeding Behaviors’ OR ‘Feeding Pattern’ OR ‘Feeding Patterns’ OR ‘Feeding Related Behavior’ OR ‘Feeding-Related Behavior’ OR ‘Feeding-Related Behaviors’ OR ‘Food Habit’ OR ‘Food Habits’ OR ‘Habit Diet’ OR ‘Habit Dietary’ OR ‘Habit Eating’ OR ‘Habit Food’ OR ‘Habits Diet’ OR ‘Habits Dietary’ OR ‘Habits Eating’ OR ‘Habits Food’ OR ‘Pattern Feeding’ OR ‘Patterns Feeding’ OR ‘Diet Meat-Sweet’ OR ‘Diet Occidental’ OR ‘Dietary Pattern Western’ OR ‘Dietary Patterns Western’ OR ‘Diets Meat-Sweet’ OR ‘Diets Occidental’ OR ‘Diets Western’ OR ‘Meat Sweet Diet’ OR ‘Meat-Sweet Diet’ OR ‘Meat-Sweet Diets’ OR ‘Occidental Diet’ OR ‘Occidental Diets’ OR ‘Pattern Western Dietary’ OR ‘Patterns Western Dietary’ OR ‘Western Diet’ OR ‘Western Dietary Pattern’ OR ‘Western Dietary Patterns’ OR ‘Western Diets’ AND ‘Adenoma Thyroid’ OR ‘Adenomas Thyroid’ OR ‘Cancer of the Thyroid’ OR ‘Cancer of Thyroid’ OR ‘Cancer Thyroid’ OR ‘Cancers Thyroid’ OR ‘Carcinoma Thyroid’ OR ‘Carcinomas Thyroid’ OR ‘Neoplasm Thyroid’ OR ‘Neoplasms Thyroid’ OR ‘Radioiodine-Refractory Thyroid Cancer’ OR ‘Thyroid Adenoma’ OR ‘Thyroid Adenomas’ OR ‘Thyroid Cancer’ OR ‘Thyroid Cancers’ OR ‘Thyroid Carcinoma’ OR ‘Thyroid Carcinomas’ OR ‘Thyroid Neoplasm’* | *10/09/2023* |
| *LILACS*  *(BVS)* | *(Behavior, Eating) OR (Behavior, Feeding) OR (Behavior, Feeding-Related) OR (Behaviors, Eating) OR (Behaviors, Feeding) OR (Diet Habit) OR (Diet Habits) OR (Dietary Habit) OR (Dietary Habits) OR (Eating Behavior) OR (Eating Behaviors) OR (Eating Habit) OR (Eating Habits) OR (Feeding Behaviors) OR (Feeding Pattern) OR (Feeding Patterns) OR (Feeding Related Behavior) OR (Feeding-Related Behavior) OR (Feeding-Related Behaviors) OR (Food Habit) OR (Food Habits) OR (Habit, Diet) OR (Habit, Dietary) OR (Habit, Eating) OR (Habit, Food) OR (Habits, Diet) OR (Habits, Dietary) OR (Habits, Eating) OR (Habits, Food) OR (Pattern, Feeding) OR (Patterns, Feeding) OR (Diet, Meat-Sweet) OR (Diet, Occidental) OR (Dietary Pattern, Western) OR (Dietary Patterns, Western) OR (Diets, Meat-Sweet) OR (Diets, Occidental) OR (Diets, Western) OR (Meat Sweet Diet) OR (Meat-Sweet Diet) OR (Meat-Sweet Diets) OR (Occidental Diet) OR (Occidental Diets) OR (Pattern, Western Dietary) OR (Patterns, Western Dietary) OR (Western Diet) OR (Western Dietary Pattern) OR (Western Dietary Patterns) OR (Western Diets) AND (Adenoma, Thyroid) OR (Adenomas, Thyroid) OR (Cancer of the Thyroid) OR (Cancer of Thyroid) OR (Cancer, Thyroid) OR (Cancers, Thyroid) OR (Carcinoma, Thyroid) OR (Carcinomas, Thyroid) OR (Neoplasm, Thyroid) OR (Neoplasms, Thyroid) OR (Radioiodine-Refractory Thyroid Cancer) OR (Thyroid Adenoma) OR (Thyroid Adenomas) OR (Thyroid Cancer) OR (Thyroid Cancers) OR (Thyroid Carcinoma) OR (Thyroid Carcinomas) OR (Thyroid Neoplasm)* | *10/09/2023* |
| ***Database*** | **Complementary Search Strategy** | **Date** |
| *Pubmed (Medline)* | *(("Diet"[Mesh]) OR (Diets) OR ("Food"[Mesh]) OR (Foods) OR ("Diet, Food, and Nutrition"[Mesh]) OR ("Nutritive Value"[Mesh]) OR (Value*, Nutri*) OR (Availability, Biologic* Nutri*) OR (Biologic* Nutri* Availability) OR (Availability, Nutri* Biologic*) OR (Nutri* Biologic* Availability) OR (Biologic* Availability, Nutri*) OR (Nutri* Value*) OR (Value*, Nutrition) OR (Nutri* Availability) OR (Availability, Nutri*) OR (Nutri* Availability, Biologic*) OR (Nutri* Quality) OR (Quality, Nutri*) OR (Nutri* Food* Quality) OR (Food* Quality, Nutri*) OR (Quality, Nutri* Food*) OR (Nutri* Quality) OR (Quality, Nutri*)) AND (("Thyroid Neoplasms"[Mesh]) OR (Neoplasm*, Thyroid*) OR (Thyroid* Neoplasm*) OR (Thyroid* Carcinoma*) OR (Carcinoma*, Thyroid*) OR (Cancer* of the Thyroid*) OR (Cancer* of Thyroid*) OR (Thyroid* Cancer*) OR (Cancer*, Thyroid*) OR (Thyroid* Adenoma*) OR (Adenoma*, Thyroid*))* | *18/01/2025* |
| *Embase (Elsevier)* | *('diet'/exp OR diet OR 'diets'/exp OR diets OR 'food'/exp OR 'food' OR 'foods' OR 'diet, food, and nutrition'/exp OR 'diet, food, and nutrition' OR 'nutritive value'/exp OR 'value*, nutri*' OR 'availability, biologic* nutri*' OR 'biologic* nutri* availability' OR 'availability, nutri* biologic*' OR 'nutri* biologic* availability' OR 'biologic* availability, nutri*' OR 'nutri* value*' OR 'value*, nutrition' OR 'nutri* availability' OR 'availability, nutri*' OR 'nutri* availability, biologic*' OR 'nutri* food* quality' OR 'food* quality, nutri*' OR 'quality, nutri* food*' OR 'nutri* quality' OR 'quality, nutri*') AND ('thyroid neoplasms'/exp OR 'neoplasm*, thyroid*' OR 'thyroid* neoplasm*' OR 'thyroid* carcinoma*' OR 'carcinoma*, thyroid*' OR 'cancer* of the thyroid*' OR 'cancer* of thyroid*' OR 'thyroid* cancer*' OR 'cancer*, thyroid*' OR 'thyroid* adenoma' OR 'adenoma*, thyroid*')* | *18/01/2025* |
| *Scopus (Elsevier)* | *‘Diet’ OR (Diets) OR ‘Food’ OR (Foods) OR ‘Diet, Food, and Nutrition’ OR ‘Nutritive Value’ OR (Value*, Nutri*) OR (Availability, Biologic* Nutri*) OR (Biologic* Nutri* Availability) OR (Availability, Nutri* Biologic*) OR (Nutri* Biologic* Availability) OR (Biologic* Availability, Nutri*) OR (Nutri* Value*) OR (Value*, Nutrition) OR (Nutri* Availability) OR (Availability, Nutri*) OR (Nutri* Availability, Biologic*) OR (Nutri* Quality) OR (Quality, Nutri*) OR (Nutri* Food* Quality) OR (Food* Quality, Nutri*) OR (Quality, Nutri* Food*) OR (Nutri* Quality) OR (Quality, Nutri*) AND "Thyroid Neoplasms" OR (Neoplasm*, Thyroid*) OR (Thyroid* Neoplasm*) OR (Thyroid* Carcinoma*) OR (Carcinoma*, Thyroid*) OR (Cancer* of the Thyroid*) OR (Cancer* of Thyroid*) OR (Thyroid* Cancer*) OR (Cancer*, Thyroid*) OR (Thyroid* Adenoma*) OR (Adenoma*, Thyroid*)* | *18/01/2025* |
| *Web of Science* | *((((((ALL=(Diet OR Diets)) OR ALL=(Food OR Foods)) OR ALL=(Diet, Food, and Nutrition)) OR ALL=(Nutritive Value OR Value*, Nutri* OR Availability, Biologic* Nutri* OR Biologic* Nutri* Availability OR Availability, Nutri* Biologic* OR Nutri* Biologic* Availability OR Biologic* Availability, Nutri* OR Nutri* Value* OR Value*, Nutrition OR Nutri* Availability OR Availability, Nutri* OR Nutri* Availability, Biologic* OR Nutri* Quality OR Quality, Nutri* OR Nutri* Food* Quality OR Food* Quality, Nutri* OR Quality, Nutri* Food* OR Nutri* Quality OR Quality, Nutri*)))) AND ALL=((Thyroid Neoplasms OR Neoplasm*, Thyroid* OR Thyroid* Neoplasm* OR Thyroid* Carcinoma* OR Carcinoma*, Thyroid* OR Cancer* of the Thyroid* OR Cancer* of Thyroid* OR Thyroid* Cancer* OR Cancer*, Thyroid* OR Thyroid* Adenoma* OR Adenoma*, Thyroid*))* | *18/01/2025* |
| *LILACS*  *(BVS)* | *((mh: "Dieta" OR ("Diet") OR ("Dieta") OR ("Diets") OR mh:G07.203.650.240$ OR mh:SP6.090.324$) OR (mh: "Comida" OR ("Food") OR ("Comida") OR ("Foods") OR mh:G07.203.300$ OR mh:J02.500$ ) OR (mh:"Diet, Food, and Nutrition" OR mh:G07.203$) OR (mh: "Valor Nutritivo" OR ("Nutritive Value") OR ("Valor Nutritivo") OR ("Value$, Nutri$") OR ("Availability, Biologic$ Nutri$") OR ("Biologic$ Nutri$ Availability") OR ("Availability, Nutri$ Biologic$") OR ("Nutri$ Biologic$ Availability") OR ("Biologic$ Availability, Nutri$") OR ("Nutri$ Value$") OR ("Value$, Nutrition") OR ("Nutri$ Availability") OR ("Availability, Nutri$") OR ("Nutri$ Availability, Biologic$") OR ("Nutri$ Quality") OR ("Quality, Nutri$") OR ("Nutri$ Food$ Quality") OR ("Food$ Quality, Nutri$") OR ("Quality, Nutri$ Food$") OR ("Nutri$ Quality") OR ("Quality, Nutri$") OR mh:G07.203.650.660$ OR mh:J01.576.423.850.730.750$ OR mh:N06.850.601.750$ OR mh:SP6.090.324.215.401.611$)) AND (mh: "Neoplasias da Glândula Tireoide" OR ("Thyroid Neoplasms") OR ("Neoplasias de la Tiroides") OR ("Neoplasm$, Thyroid$") OR ("Thyroid$ Neoplasm$") OR ("Thyroid$ Carcinoma$") OR ("Carcinoma$, Thyroid$") OR ("Cancer$ of the Thyroid$") OR ("Cancer$ of Thyroid$") OR ("Thyroid$ Cancer$") OR ("Cancer$, Thyroid$") OR ("Thyroid$ Adenoma$") OR ("Adenoma$, Thyroid$") OR mh:C04.588.322.894$ OR mh:C04.588.443.915$ OR mh:C19.344.894$ OR mh:C19.874.788$)* | *18/01/2025* |

**Supplementary Table S2 – Data extraction form**

**Supplementary Table S3 – Newcastle-Ottawa Scale (NOS) Classification**

| First author / year | Selection | | | | | Comparability | | Exposure/Outcome | | | Total | |  |
| --- | --- | --- | --- | --- | --- | --- | --- | --- | --- | --- | --- | --- | --- |
|  | **1** | **2** | **3** | **4** | **1 ou 2** | | **1** | | **2** | **3** | |  | |
| Franceschi et al. (1989) | 1 | 1 | 0 | 1 | 2 | | 0 | | 1 | 1 | | 6 | |
| Kolonel et al. (1990) | 1 | 1 | 1 | 0 | 2 | | 0 | | 1 | 0 | | 6 | |
| Negri et al. (1991) | 1 | 1 | 0 | 1 | 2 | | 0 | | 1 | 1 | | 7 | |
| Glattre et al. (1992) | 1 | 1 | 1 | 1 | 2 | | 0 | | 1 | 0 | | 7 | |
| Wingren et al. (1993) | 0 | 1 | 1 | 1 | 2 | | 0 | | 1 | 1 | | 7 | |
| Takezaki et al. (1996) | 1 | 1 | 0 | 1 | 1 | | 0 | | 1 | 0 | | 5 | |
| Galanti et al. (1997) | 1 | 1 | 1 | 1 | 2 | | 0 | | 1 | 0 | | 7 | |
| Fioretti et al. (1999) | 1 | 1 | 1 | 1 | 2 | | 0 | | 1 | 1 | | 8 | |
| Frentzel-Beyme e Helmert. (2000) | 1 | 1 | 1 | 0 | 2 | | 0 | | 1 | 0 | | 6 | |
| Memon et al. (2002) | 1 | 1 | 1 | 0 | 2 | | 0 | | 1 | 1 | | 7 | |
| Horn-Ross et al. (2002) | 1 | 1 | 1 | 0 | 2 | | 0 | | 1 | 1 | | 7 | |
| Markaki et al. (2003) | 1 | 1 | 0 | 1 | 1 | | 1 | | 1 | 0 | | 6 | |
| Haselkori et al. (2003) | 1 | 1 | 1 | 0 | 2 | | 0 | | 1 | 1 | | 7 | |
| Randi et al. (2008) | 1 | 1 | 0 | 1 | 2 | | 0 | | 1 | 0 | | 6 | |
| Truong et al. (2010) | 1 | 1 | 1 | 1 | 2 | | 0 | | 1 | 1 | | 8 | |
| Jung et al. (2012) | 1 | 1 | 0 | 1 | 2 | | 0 | | 1 | 0 | | 6 | |
| Przybylik-Mazurek et al. (2012) | 1 | 1 | 1 | 1 | 1 | | 0 | | 1 | 0 | | 6 | |
| Clero et al. (2012) | 1 | 1 | 1 | 0 | 2 | | 0 | | 1 | 0 | | 6 | |
| Michikawa et al. (2012) | 0 | 1 | 1 | 1 | 2 | | 1 | | 1 | 1 | | 8 | |
| Aschebrook-Kilfoy et al. (2012) | 1 | 1 | 1 | 1 | 2 | | 1 | | 1 | 1 | | 9 | |
| Xiao et al. (2014) | 1 | 1 | 1 | 0 | 1 | | 0 | | 1 | 1 | | 6 | |
| Wie et al. (2014) | 0 | 1 | 1 | 1 | 1 | | 0 | | 1 | 0 | | 5 | |
| Yoo et al. (2016) | 1 | 1 | 0 | 1 | 2 | | 0 | | 1 | 0 | | 6 | |
| Zamora-Ros et al. (2018) | 1 | 1 | 1 | 1 | 2 | | 0 | | 1 | 1 | | 8 | |
| Sangsefidi et al. (2019) | 1 | 1 | 1 | 1 | 2 | | 0 | | 1 | 0 | | 7 | |
| Paquet et al. (2020) | 1 | 1 | 1 | 1 | 2 | | 0 | | 1 | 1 | | 8 | |
| Fiore et al. (2020) | 1 | 1 | 0 | 1 | 2 | | 0 | | 1 | 1 | | 7 | |
| Liang et al. (2020) | 1 | 1 | 1 | 1 | 2 | | 1 | | 1 | 0 | | 8 | |
| Choi et al. (2021) | 1 | 1 | 0 | 2 | 2 | | 1 | | 1 | - | | 8 | |
| Amiri et al. (2021) | 1 | 1 | 1 | 1 | 1 | | 0 | | 1 | 1 | | 6 | |
| Fiore et al. (2021) | 1 | 1 | 0 | 1 | 2 | | 0 | | 1 | 1 | | 7 | |
| Manman et al. (2021) | 1 | 1 | 0 | 1 | 2 | | 0 | | 1 | 0 | | 6 | |
| Parad et al. (2021) | 1 | 1 | 1 | 1 | 2 | | 0 | | 1 | 0 | | 7 | |
| Tanitame et al. (2022) | 1 | 1 | 1 | 1 | 1 | | 1 | | 1 | 1 | | 8 | |
| Llahar et al. (2022) | 1 | 1 | 1 | 1 | 2 | | 0 | | 1 | 0 | | 7 | |
| Jin et al. (2022) | 1 | 1 | 0 | 0 | 2 | | 0 | | 1 | 1 | | 6 | |
| Vasconcelos et al. (2022) | 0 | 1 | 1 | 2 | 0 | | 1 | | 0 | -- | | 5 | |
| Xia et al. (2022) | 1 | 1 | 0 | 1 | 2 | | 0 | | 1 | 0 | | 6 | |
| Lécuyer et al. (2022) | 1 | 1 | 1 | 0 | 2 | | 0 | | 1 | 1 | | 7 | |
| Nguyen et al. (2023) | 1 | 1 | 1 | 0 | 2 | | 1 | | 0 | 1 | | 7 | |
| La Greca et al. (2023) | 1 | 1 | 1 | 1 | 2 | | 0 | | 1 | 1 | | 8 | |
| Binobead et al. (2023) | 1 | 1 | 0 | 1 | 1 | | 0 | | 1 | 0 | | 5 | |
| Zamora-Ros et al. (2023) | 1 | 1 | 1 | 1 | 2 | | 1 | | 1 | 1 | | 9 | |
| Kwon et al. (2024) | 1 | 1 | 0 | 0 | 2 | | 0 | | 1 | 1 | | 6 | |

**Supplementary Figure S1_Distribution of thyroid cancer publications by continent and number of cases over time.**

Number of cases

Year of publication

### Source: Authors, 2025.
